# Local level estimates of food, drink and tobacco expenditure for Great Britain

**DOI:** 10.1038/s41597-019-0064-z

**Published:** 2019-05-13

**Authors:** William H. M. James, Nik Lomax, Mark Birkin

**Affiliations:** School of Geography and Leeds Institute for Data Analytics, University of Leeds, Woodhouse Lane, Leeds, West Yorkshire LS2 9JT UK

**Keywords:** Geography, Agriculture, Environmental impact, Nutrition, Obesity

## Abstract

We present expenditure estimates for 106 product categories across Great Britain for the years 2008–2016. Estimates are at the Local Authority District level (n = 380) and the categories cover all food, drink and tobacco commodities. Reliable, local level expenditure estimates are crucial for understanding broader market trends, assessing economic stability and for projections. This is especially important for commodities such as alcohol, tobacco and unhealthy foods due to their role in the prevalence of non-communicable diseases. There has been relatively little research into local area spatial patterns of expenditure, with existing estimates often of insufficient resolution for informing planning decisions. We use spatial microsimulation to create an archive of expenditure datasets. This was achieved by linking socio-demographic foundations with detailed datasets on individual expenditure. Whilst initially developed to aid investigations into sociodemographic trends in the meat industry, the data have reuse potential in a number of disciplines, including public health, economics, retail geography and environmental management. The framework could be applied to other regions with appropriate data.

## Abstract (summary)

**Table:** 

Design Type(s)	data integration objective • modeling and simulation objective • population data analysis objective
Measurement Type(s)	expenditure
Technology Type(s)	computational modeling technique
Factor Type(s)	temporal_interval
Sample Characteristic(s)	Homo sapiens • United Kingdom • anthropogenic habitat

Machine-accessible metadata file describing the reported data (ISA-Tab format)

## Background & Summary

Over the past 50 years, the UK has experienced major shifts in dietary patterns due to changes in agricultural practice, trade policies and food industry marketing^1^. Further changes may be on the horizon in the context of a UK exit from the European Union^2^. Against this backdrop of continuous national level change, there is substantial local level variability in food consumption and expenditure patterns, which has been attributed to spatial variations in factors including socio-economic status^3–5^ and demographics^6^. These changes are reflected by individual expenditure patterns as surveyed annually by the Living Costs and Food Survey^7–15^, with corresponding results published to the regional level across the UK^16^ (i.e. 12 geographical zones). To help understand the local level variability of expenditure, and to form a baseline for future projections, we present an open access archive of expenditure datasets for Great Britain for the years 2008 to 2016. Each annual dataset consists of an expenditure estimate for 106 food, drink and tobacco categories for every Local Authority District (LAD) (n = 380).

Robust estimates of local level spatial patterns of food and drink expenditure are crucial for understanding broader trends, for assessing market stability and for future projections. It has long been argued that the most powerful theoretical models for explaining human behaviour operate at the individual person level^17^, with emergent higher-level properties giving the best opportunity to understand the entire system at all levels. Reliable and detailed information on the spatial distribution of food, drink and tobacco expenditure is also key for research in the fields of public health, environmental impact and retail geography. This importance is highlighted by the prevalence of non-communicable diseases such as cardiovascular disease, cancer and diabetes which currently account for 70% of all deaths worldwide and 90% in the UK^18^. As these diseases share key modifiable behavioural risk factors such as tobacco use, unhealthy diet and the harmful use of alcohol, it is clear that understanding expenditure patterns associated with key commodities is of great value for public health research. There is also an increased awareness of the environmental impact of food production, with livestock production responsible for 14.5% of all anthropogenic greenhouse gas emissions in 2004^19^, whilst 71.2% of deforestation in South America between 1990 and 2005 was for conversion to pasture^20^.

There has been relatively little research into local area spatial patterns of food and drink expenditure in the UK. Whilst expenditure data is routinely collected by companies and organisations, these are seldom open-access, often at a coarse spatial resolution and only provide a snapshot of specific products or socio-economic groups. In the UK, comprehensive estimates of food and drink expenditure are published annually by the Department for Environment, Food & Rural Affairs (DEFRA)^16^, representative of the population. However, these data are only available at the regional level (12 geographical zones) and as such are not at a sufficient resolution for informing planning decisions related to public health infrastructure, retail or the environment at the local level. For example, concerns over access to healthy foods^21^ cannot be assessed using regional level data. Furthermore, there is little information on associated individual level socio-demographics, which have been shown to be strongly linked to expenditure^3^.

This study aims to bridge the identified data gap between the published regional level estimates of expenditure^16^ and known drivers of local level variation^3–6^ by producing local area level datasets of expenditure using the technique of spatial microsimulation. Increasing computational efficiency and falling costs combined with the improved availability of survey microdata have increased the ability to produce such datasets^22^. As such, using the code developed by Lovelace and Dumont^23^, an archive of expenditure datasets has been created. This process used the most recent census and survey microdata available to the authors at the time of writing, alongside a range of geospatial datasets.

We believe spatial microsimulation techniques of the type described in this paper hold great potential benefits for a range of disciplines including economics, retail geography and public health. Whilst this study focusses on Great Britain, the framework here could be applied to any location with the appropriate data sources.

## Methods

### Spatial microsimulation

**Fig. 1:**
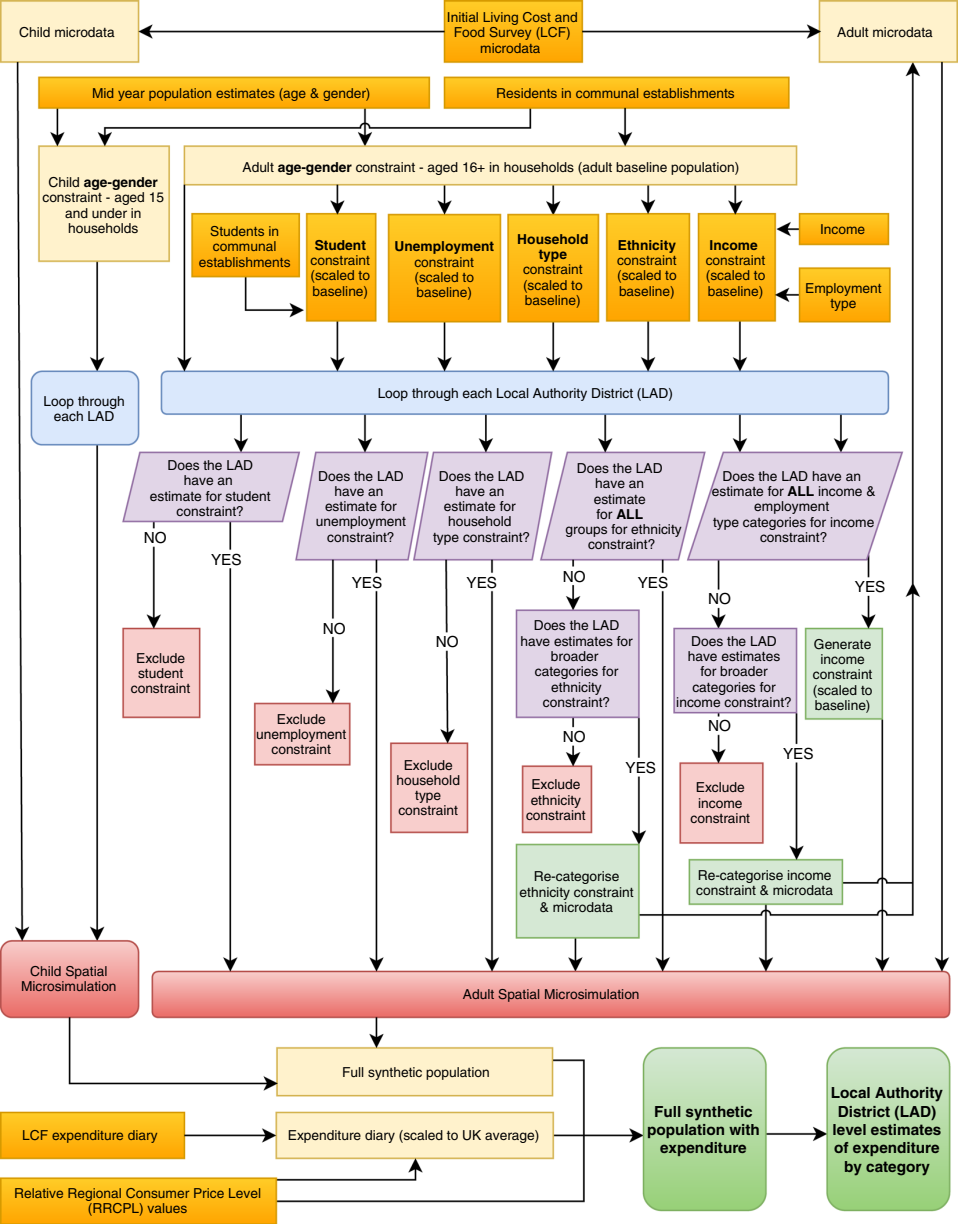
Schematic diagram of the expenditure estimation framework.

This study uses spatial microsimulation to generate expenditure estimates under the framework shown in Fig. 1. Spatial microsimulation involves ‘*the creation*, *analysis and modelling of individual level data allocated to geographic zones*’^23^, and has been used in the fields of health care demand^24^, educational attainment^25^, commuting patterns^26^, and population projections^27^ amongst others. For a comprehensive overview of the microsimulation process the reader is directed to Birkin and Clarke^28^ and a guide to implementation can be found in Lomax and Smith^29^.

As with most spatial microsimulation models, the input data for this study consists of microdata – a non-geographical individual level dataset – and constraint tables, which provide aggregate counts for each geographical zone (LAD). The framework is split into two separate microsimulation models as shown in Fig. 1; a comprehensive ‘adult’ model for those aged 16 and over and a ‘child’ model for those aged 15 and under. Once each of the models have completed, the results are merged to generate a full synthetic population and consequently LAD level estimates of expenditure.

Specifically, this study employs Iterative Proportional Fitting (IPF), implemented within the R programming environment (https://www.r-project.org). IPF works by adjusting a large array of weights - rows corresponding to individuals and columns corresponding to the geographic zones (e.g. LADs) - iteratively, to maximise the fit between simulated and known (e.g. census/survey) data. The mathematics of IPF are covered by Fienberg^30^ a guide to implementation is provided in Lomax and Norman^31^ whilst the code used here for implementing IPF in R was developed by Lovelace and Dumont^23^.

### Microdata - Living Cost and Food Survey (LCF)

Microdata are taken from the Living Cost and Food Survey (LCF), the most comprehensive survey on household spending in the UK, covering approximately 12,000 respondents from 6,000 households each year. The LCF is carried out by the Office for National Statistics (ONS) and has been running in its current format since 2008. The LCF is designed to be representative of people living in households in the UK, using a multi-stage stratified random sample with clustering approach. The survey is weighted to compensate for non-response and also to ensure the sample distribution matches the population distribution in terms of region, age group and sex. The LCF runs continuously throughout the year to avoid seasonal variation^32^.

The LCF comprises an expenditure diary detailing purchases over a two-week period and an interview covering socio-demographic characteristics, income and regular items of household expenditure. Respondents are required to record all expenditure over the two-week period (regardless of outlet), thus providing a comprehensive account of household expenditure. Commodities recorded in the LCF diary (and consequently in this study) are grouped by category, based on The Classification of Individual Consumption by Purpose (COICOP) coding framework. COICOP groups products into homogenous categories for which food, drink and tobacco constitute 106 separate groups. Categories may define a specific product (e.g. 1.1.6.2.1 = Bananas – fresh) or a homogenous group of products (e.g. 1.1.1.4.1 = Cakes and puddings). The framework structure also allows easy aggregation to higher levels (e.g. 01.1.2.5 = Dried, salted or smoked meat and edible meat offal; 01.1.2 = Meat; and 01.1 = Food). The full list of 106 food, drink and tobacco expenditure codes used in this study can be found in Supplementary File 1. The 2016–2017 LCF survey reported some commodities (specifically those consumed away from home) only to an aggregate level, resulting in fewer categories for our 2016 dataset (n = 80). These aggregated categories are included in Supplementary File 1. Whilst grouping products in this manner may mean that analysis related to specific products is restricted, the 106 categories provide sufficient detail for most applications. Whilst various other coding frameworks are available, COICOP was specifically developed by the United Nations Statistics Division to analyse individual expenditures, and was therefore adopted by the ONS for use in the LCF. Datasets presented in this study can be directly compared with others which use the COICOP framework, whilst the detailed descriptions provided in Supplementary File 1 allows cross-referencing with alternative frameworks if required.

The LCF is geocoded at a coarse level, detailing which of the 12 government regions each individual resides in (Scotland, Wales, Northern Ireland, South East, London, North West, East of England, West Midlands, South West, Yorkshire and the Humber, East Midlands, North East). As discussed previously, this is insufficient for informing planning decisions related to public health infrastructure, retail or the environment at the local level. Whilst it would be technically possible to constrain the microsimulation model using these data (i.e. only individuals sampled in the South West region of England from the LCF would be able to be assigned to LADs in the South West region), this would result in a much-reduced sampling pool insufficient for spatial microsimulation. As such, no initial geographical constraints are used in the microsimulation model although the regional information is used to account for relative regional price levels and for model validation purposes, as discussed in due course.

It should be noted that whilst the LCF survey includes individuals from Northern Ireland, insufficient constraint variables were available for microsimulation within Northern Ireland (see below). As such, whilst microsimulation outputs presented in this paper are restricted to Great Britain, individuals from Northern Ireland (from the LCF) are included within the sampling pool and may be allocated to any LAD in Great Britain if their socio-demographic characteristics are appropriate.

### Formatting the survey microdata

The LCF contains a wealth of information, much of which is not required for the purposes of this study and can thus be discarded. As the microsimulation process requires common variable classes for the microdata and corresponding constraint dataset, re-formatting is required to generate the appropriate classes. Table 1 lists the LCF variables used in this study and their categorisation. Table 2 provides an example extract of the formatted socio-demographic microdata, and Table 3 shows an example of the diary information.

**Table 1:** LCF microdata fields and classes.

Variable	Description	Values
ID	Individual identifier code	e.g. 3.2 (number before decimal point indicates household, number after indicates the individual within the household). This is used to link with the expenditure diary (Table 3)
Region	Which UK region the individual is from	South East
		London
		Scotland
		Wales
		Northern Ireland
		West Midlands
		South West
		North East
		North West
		Eastern
		East Midlands
		Yorkshire
Age & sex	Age group and sex	Female, aged 0–9 years
		Female, aged 10–15 years
		Female, aged 16–24 years
		Female, aged 25–34 years
		Female, aged 35–49 years
		Female, aged 50–64 years
		Female, aged 65–74 years
		Female, aged 75+ years
		Male, aged 0–9 years
		Male, aged 10–15 years
		Male, aged 16–24 years
		Male, aged 25–34 years
		Male, aged 35–49 years
		Male, aged 50–64 years
		Male, aged 65–74 years
		Male, aged 75+ years
Ethnicity	Ethnicity	Black
		White
		Mixed
		Other
Unemployed	Whether or not is unemployed	Unemployed
		Not-unemployed
Student	Whether or not a full-time student	Student
		Not-student
Gross wage	Gross weekly wage (£) (employees only)	e.g. £542.56
Employment type	Type of employment	Employee
		Self-employed
		Other
Household type	Type of household – grouped age of individual followed by number/age of dependent children	16_24_dep_n (Aged 16–24, no dependent children in household)
		16_24_dep_y (Aged 16–24, dependent children in household)
		25_34_dep_n (Aged 25–34, no dependent children in household)
		25_34_dep_y_0_4 (Aged 25–34, youngest dependent child aged 0–4)
		25_34_dep_y_5_10 (Aged 25–34, youngest dependent child aged 5–10)
		25_34_dep_y_11_pl (Aged 25–34, youngest dependent child aged 11+)
		35_54_dep_n (Aged 35–54, no dependent children in household)
		35_54_dep_y_0_4 (Aged 35–54, youngest dependent child aged 0–4)
		35_54_dep_y_5_10 (Aged 35–54, youngest dependent child aged 5–10)
		35_54_dep_y_11_pl (Aged 35–54, youngest dependent child aged 11+)
		55_64_mph_dep_n (Aged 55–64, no dependent children in household)
		55_64_sph (Aged 55–64, single person household)
		55_74_dep_y (Aged 55–74, dependent children in household)
		65_74_mph_dep_n (Aged 65–74, multiple person household, no dependent children)
		65_74_sph (Aged 65–74, single person household)
		75_pl_mph (Aged 75+, multiple person household)
		75_pl_sph (Aged 75+, single person household)

**Table 2:** Sample formatted microdata. See Table 1 for a description of variables.

ID	Expenditure region	Age sex	Ethnicity	Unemployed	Student	Gross weekly wage (£)	Employment type	Household type
9.1	South East	M_50_64	white	N	N	1052.97	Employee	35_54_dep_n
9.2	South West	F_25_34	mixed	N	Y	0	Employee	25_34_dep_n
4583.3	London	F_16_24	mixed	N	N	30	Employee	16_24_dep_n
3793.1	London	F_25_34	white	N	N	0	Other	25_34_dep_y_5_10
5194.2	Scotland	F_25_34	white	N	N	273.2	Employee	25_34_dep_n
2426.2	Wales	M_35_49	white	N	N	350.8	Employee	A_35_54_dep_y_0_4
3061.2	Wales	F_50_64	white	N	N	0	Self-employed	A_55_64_mph_dep_n

**Table 3:** Sample expenditure diary structure.

ID	COICOP expenditure code	Weekly expenditure (£)
2.2	11.7.1.1.5	23.08
2.2	11.1.3.1.5	25
8.1	1.1.1.2.2	0.52
8.1	1.1.8.4.1	0.5
8.1	1.2.2.2.1	0.375

From 2015 onwards the LCF reporting window moved from a calendar year (January to December) to a financial year (April to March). To maintain consistency of our datasets, we use a calendar year throughout (i.e. our 2015 dataset represents LCF data from January 2015 to December 2015). As the LCF details when each survey was completed during the year, we achieve this by removing and appending records from each year as appropriate. The 2015–2016 LCF survey also includes additional records from January to March 2015, making it possible to construct a seamless data series.

### Constraint variables

As with other microsimulation applications, the model presented here is underpinned by the assumption that the target variable (expenditure) is associated with the geographical constraint variables. Constraint variables were chosen following the guidelines of Lovelace and Dumont^23^, based upon relevance to the target variable (expenditure) and data availability. Table 4 details the constraint variables selected, the source datasets and their temporal coverage. The microsimulation is split into two separate sub-routines: a comprehensive ‘adult’ microsimulation model for those aged 16 and over and a simpler ‘child’ microsimulation model for those aged under 16. This is because many of the variables are not available and/or not applicable for those under the age of 16 (e.g. unemployment). As noted previously, many of the constraints listed in Table 4 are not available for Northern Ireland and as a result the microsimulation presented here was restricted to Great Britain.

**Table 4:** Constraint variables and source datasets for the adult and child microsimulation models. *The age and sex constraint forms the baseline population to which all other constraints are scaled to.

Model	Constraint	Source dataset(s)	Sample description	Temporal coverage
Adult	Age and sex, household residents (cross tabulated)*	ONS Mid-year population estimates - local authority based by single year of age (http://www.nomisweb.co.uk)	All residents aged 16 and over	Annual 2008–2016
		Census 2011 Table LC1105EW - Residence type by sex by age. (http://www.nomisweb.co.uk)	Residents living in communal establishments aged 16+ (England and Wales)	2011
		National Records of Scotland. Scotland’s Census 2011 - Table DC4414SCca - Communal establishment type by type of resident by sex by age. (http://www.scotlandscensus.gov.uk)	Residents living in communal establishments aged 16+ (Scotland)	2011
Adult	Ethnicity	ONS Annual Population Survey (http://www.nomisweb.co.uk)	Ages 16+. Excludes communal establishment residents other than those in NHS housing or student halls.	Annual 2008–2016
Adult	Student	ONS Annual Population Survey (http://www.nomisweb.co.uk)	Ages 16+. Excludes communal establishment residents other than those in NHS housing or student halls.	Annual 2008–2016
		Census 2011 Table LC4411EW - Student accommodation by age (http://www.nomisweb.co.uk)	Students living in communal establishments, ages 16+ (England and Wales)	2011
		Scotland’s Census 2011 - Table DC4414SCca - Communal establishment type by type of resident by sex by age (http://www.scotlandscensus.gov.uk)	Students living in communal establishments, ages 16+ (Scotland)	2011
Adult	Unemployment	ONS Model-based estimates of unemployment (http://www.nomisweb.co.uk)	Ages 16+. Excludes communal establishment residents other than those in NHS housing or student halls.	Annual 2008–2016
Adult	Household type	Census 2011 Table QS110UK - Adult life-stage (alternative adult definition) (http://www.nomisweb.co.uk)	Household residents, aged 16+	2011
Adult	Income	ONS Annual Survey of Hours and Earnings (http://www.nomisweb.co.uk)	Ages 16+. Gross weekly pay - sample of employee jobs taken from HM Revenue and Customs PAYE records.	Annual 2008–2016
		ONS Annual Population Survey (http://www.nomisweb.co.uk)	Ages 16+. Count of employees and self-employed. Excludes communal establishment residents other than those in NHS housing or student halls.	Annual 2008–2016
Child	Age and sex, household residents (cross tabulated)*	ONS mid-year population estimates - local authority based by single year of age (http://www.nomisweb.co.uk)	All residents aged 16+	Annual 2008–2016
		Census 2011 Table LC1105EW - Residence type by sex by age (http://www.nomisweb.co.uk)	Residents living in communal establishments aged 15 and under (England and Wales)	2011
		Scotland’s Census 2011 - Table DC4414SCca - Communal establishment type by type of resident by sex by age (http://www.scotlandscensus.gov.uk)	Residents living in communal establishments aged 15 and under (Scotland)	2011

### Constructing the baseline population

Microsimulation requires the baseline population of each constraint (i.e. the total number of people in each zone) to correspond to the population from which the microdata has been sampled. For the LCF, this is all people living in households aged 16 and over (for the adult model) or aged 15 and under (for the child model). The IPF algorithm also requires the baseline population to be identical across all constraint variables. To meet these requirements, our baseline population for each year is taken from the Office for National Statistics mid-year population estimates, with residents living in communal establishments removed to result in only residents living in households. All other constraints are scaled to this baseline population, as described by Lovelace and Dumont^23^. Table 5 shows an extract of the final 2008 age-sex constraint table (household residents only) for three local authorities.

**Table 5:** Sample of the age-sex constraint for 2008 (household residents only). (Note table is for illustrative purposes only and therefore does not display all age groups).

LA code	F_16_24	F_25_34	F_35_49	F_50_64	F_65_74	F_75_pl	…..	M_75_pl	Total (16+) Baseline
E08000002	9853	11541	21164	16872	8063	7124	…..	4718	144258
E08000003	39476	44085	43929	28251	13180	13758	…..	8531	364549
E08000004	13280	13949	24043	19388	9031	8009	…..	5124	170259

With counts of communal establishment residents only available for the year 2011, we assume that this population is unchanged throughout the study years (2008 to 2016). This is a reasonable assumption, as communal establishment populations are usually fairly stable in terms of their size and demographic structure. For example, an elderly care home will contain a similar group of individuals from year to year. This stability is recognised by the ONS, who treats communal establishment populations as a different and more stable group to the household population when producing the mid-year estimates^33^. Furthermore, any deviation from the 2011 counts will have a negligible impact on the model output as communal establishment residents account for a small proportion of the overall population - just 1.7% in 2011^34^.

### Formatting the ethnicity constraint

Annual estimates of the number of people aged 16 and over per ethnic group for each LAD are taken from the Annual Population Survey (APS) (Table 4). These data are categorised to correspond to the LCF microdata classes (Table 1) and scaled to the baseline population. An extract of the final 2008 dataset is shown in Table 6. As the APS sample already excludes most communal residents, we assume that the proportions of each ethnic group is consistent between the baseline population and the APS sample.

**Table 6:** Extract from the student, unemployment and ethnicity constraints for 2008. Counts are for those aged 16 and over living in a household.

LA code	Student constraint		Unemployment constraint		Ethnicity constraint			
	student	non-student	unemployed	not unemployed	white	mixed	black	other
E08000002	5745	138513	5032	139226	134004	804	1106	8344
E08000003	22054	342495	20375	344174	281654	5336	22105	55454
E08000004	8115	162144	8416	161843	139659	1405	803	28392

### Formatting the unemployment constraint

Annual estimates of the number of unemployed people aged 16 and over in each LAD are taken from ONS Model Based Estimates of Unemployment (Table 4). These data are scaled to the baseline population, with an extract of the final 2008 dataset shown in Table 6. The unemployment estimates are derived from the Labour Force Survey, which excludes most communal establishment residents^35^. As such, we assume that the proportion of those unemployed is consistent between the baseline population and the model based estimates.

### Formatting the student status constraint

Annual estimates of the total number of students aged 16 and over are taken from the Annual Population Survey (Table 4). As the LCF microdata does not sample students who reside in halls of residence, these students are removed from the constraint estimate. This is achieved using 2011 Census estimates of the numbers of people aged 16 and over who live in student accommodation (Table 4). As these records are only available for 2011, we assume that the number of students in halls of residence remains constant throughout 2008–16, a reasonable assumption due to the transient nature of the population. These resulting data are scaled to the baseline population, with an extract of the final 2008 dataset shown in Table 6.

### Formatting the income constraint

Annual estimates of gross weekly pay (pre-tax) for each LAD is taken from the Annual Survey of Hours and Earnings (ASHE) (Table 4). This provides data on the pay levels and distribution of UK employees aged 16 and over. The ASHE is based on a sample of employee jobs taken from Her Majesty’s Revenue and Customs Pay As You Earn (PAYE) records and as such does not include those who are self-employed. The initial data is provided in terms of percentiles with data available for P10, P20, P30, P40, P50, P60, P70 and P80 (Table 7). Each percentile indicates the value below which a given percentage of the observations fall; for example a P20 value of £219.80 indicates that 20% of the sample has an income of less than £219.80.

**Table 7:** Extract of initial employee earnings dataset (ASHE) and employment type (taken from the APS) for 2008.

LA code	Gross weekly pay (percentiles) (ASHE)								Employment status (APS)		
	P10 (£)	P20 (£)	P30 (£)	P40 (£)	P50 (£)	P60 (£)	P70 (£)	P80 (£)	Employees (count)	Self-employed (count)	Other (count)
E08000002	144.0	219.8	293.0	358.7	408.3	480.5	584.7	669.9	75296	10053	58909
E08000003	90.0	161.1	237.4	288.5	342.9	402.7	473.2	556.0	168744	19342	176463
E08000004	116.2	220.7	261.4	304.6	359.9	428.5	483.5	571.6	81969	10434	77856

To make the ASHE data compatible with the microsimulation model, it is first necessary to estimate the total number of people covered by the sample. This is achieved using employment status estimates from the Annual Population Survey (Table 4). This provides estimates of the number of employees (i.e. those covered by PAYE records) per LAD (Table 7).

Once the number of persons in each category has been estimated, a constraint table is generated containing the income brackets for each LAD (in £s) and the number of employees within each category (Table 8). As with other constraints the values are scaled to match the baseline population. The same income brackets are used to categorise the LCF microdata for each individual LAD as shown in Fig. 1

**Table 8:** Extract of earnings constraint for three LADs. Note the different income brackets (in £s) for each LAD. (Note table is for illustrative purposes only and therefore does not display all income groups).

LA code	P0–P10		P10–P20		…	P60–P70		P70–P80		P80–P100		Self-employed (count)	Other (count)
	£ range	count	£ range	count	…	£ range	count	£ range	count	£ range	count		
E08000002	£0.0– £144.0	7529.6	£144.0– £219.8	7529.6	…	£480.5– £584.7	7529.6	£584.7– £669.9	7529.6	£669.9 +	15059.2	10053	58909
E08000003	£0.0– £90.0	16874.4	£90.0– £161.1	16874.4	…	£402.7– £473.2	16874.4	£473.2– £556.0	16874.4	£556.0 +	33748.8	19342	176463
E08000004	£0.0– £116.2	8196.9	£116.2– £220.7	8196.9	…	£428.5– £483.5	8196.9	£483.5– £571.60	8196.9	£571.6 +	16393.8	10434	77856

.

### Formatting the household characteristics constraint

Data on the household characteristics of each LAD is taken from the 2011 Census (Table 4). The dataset covers all individuals aged 16 and over living in a household, providing a description of household type (age and number of people and dependent children living in the household). The categories are grouped to correspond with those in the LCF microdata (Table 1). As information is available only for 2011, we assume that the proportion of each household type remains constant, being scaled to the baseline population each year. Table 9 shows an extract of the final household characteristics constraint table.

**Table 9:** Extract from final household characteristics constraint table for 2008. For description of variable names see Table 1. (Note table is for illustrative purposes only and therefore does not display all household types).

LA code	16_24_dep_n	16_24_dep_y	25_34_dep_n	25_34_dep_y_0_4	….	75_pl_mph	75_pl_sph
E08000002	8115	11208	11888	8058	….	6543	5519
E08000003	51303	27259	62712	23892	….	10259	9861
E08000004	9753	16287	12455	11719	….	6673	6512

### Child microsimulation model

For people aged under 16 years of age, a simpler microsimulation model is employed as many of the constraint variables are not applicable or not available (e.g. unemployment). A simpler model is also deemed appropriate as children contribute a negligible amount of total expenditure, accounting for just 0.78% in 2016–17 according to the Living Cost and Food Survey^15^. As with the adult model, ONS mid-year population estimates are used in conjunction with 2011 Census estimates of communal residents to create a baseline population. The child model uses an age sex constraint with age categories of 0–9 years (male), 10–15 (male), 0–9 years (female) and 10–15 (female), as shown in Fig. 1.

### Missing variables

Whilst constraints of age-sex and household type are available for all 380 local authorities across Great Britain, other constraints (students, unemployment, ethnicity and income) are unavailable for a minority of LADs due to small sample sizes or missing data. For example the Isles of Scilly have a total population of just 2,292 people (2014 estimate), meaning that some constraints would be disclosive if published. Whilst this is not deemed an issue in terms of model robustness, the model needs to be able to cope with missing data. This is achieved by dynamically adjusting the final constraint table for each LAD depending on which variables (and categories within) are available. Whilst the student and unemployment variables are binary (either available or not available), the variables of ethnicity and income may be partially complete (e.g. there may be an estimate of the number of individuals of black ethnicity but no estimate for those of mixed ethnicity). In these cases, the constraint (and microdata) is re-categorised to utilise the available data. For example, if an estimate for those of mixed ethnicity is unavailable for a particular LAD, new categories of ‘black’, ‘white’ or ‘other’ will be created.

In most circumstances a complete suite of constraints are available allowing for a full microsimulation model. As all LADs have complete age-sex and household type variables the microsimulation model will run on these as a minimum. Table 10 shows the number of LADs with each constraint available for each year.

**Table 10:** Number of LADs which have each constraint available for each year.

	Age/sex	household type	Student	Unemployment	Ethnicity (limited categories)	Ethnicity (all categories)	Income (limited categories)	Income (all categories)
2008	380	380	356	378	379	97	377	292
2009	380	380	363	378	379	107	378	334
2010	380	380	367	378	379	107	378	334
2011	380	380	364	378	379	114	378	342
2012	380	380	356	378	379	114	377	329
2013	380	380	364	378	379	121	377	332
2014	380	380	364	378	379	117	378	339
2015	380	380	358	378	379	120	377	339
2016	380	380	351	378	379	120	377	334

### Accounting for relative regional consumer price levels

It is well known that the price of goods and services varies throughout the UK^36^. In 2016 food and non-alcoholic beverages in London cost 2.2% more than the UK average whilst in Scotland they cost 0.2% below average^36^. This is a potential problem for the microsimulation model as the process allows an individual from the LCF microdata to be assigned to any LAD in Great Britain, according to the constraint variables. For example, if an individual from Scotland (from the LCF microdata) is assigned to a London LAD, their expenditure will likely be under-estimated.

To account for this, ONS Relative Regional Consumer Price Levels (RRCPLs) data^36,37^ are used to adjust expenditure values depending on their source region (from the LCF microdata) and their destination region (as assigned by the microsimulation model). Pre-microsimulation expenditure values are scaled to a ‘UK average’ price before being adjusted back to regional levels according to the region in which the microsimulation model assigns them to. The ONS provides an aggregate RRCPL value for each of the 12 regions (for all products) and provides more detailed category level values for London, Scotland, Northern Ireland and Wales. As such we use the detailed category level values where available and the aggregate value for all categories where not, as shown in Table 11 (for 2016). As RRCPL figures are not published annually, we use the closest datasets available; 2010 RRCPLs^37^ for 2008 to 2012 and 2016 RRCPLs^36^ for 2013 onwards.

**Table 11:** Regional price level relative to national price level (UK = 100), 2016. Adapted from ONS^36^. *Product category breakdown RRCPLs are not available for some regions so aggregate RRCPLs are used for all categories.

	Food and non-alcoholic beverages	Alcohol and tobacco	Restaurants and hotels
	(household consumption)	(household consumption)	
London	102.2	103	113
Scotland	99.8	99.4	100.4
Wales	100.8	102.3	95.1
Northern Ireland	99.7	98.6	98.3
South East*	101.5	101.5	101.5
Eastern*	99.8	99.8	99.8
West Midlands*	98.5	98.5	98.5
South West*	102.4	102.4	102.4
East Midlands*	99.6	99.6	99.6
North West & Merseyside*	98.8	98.8	98.8
North East*	98.8	98.8	98.8
Yorkshire and the Humber*	97.7	97.7	97.7

### GIS expenditure datasets

**Fig. 2:**
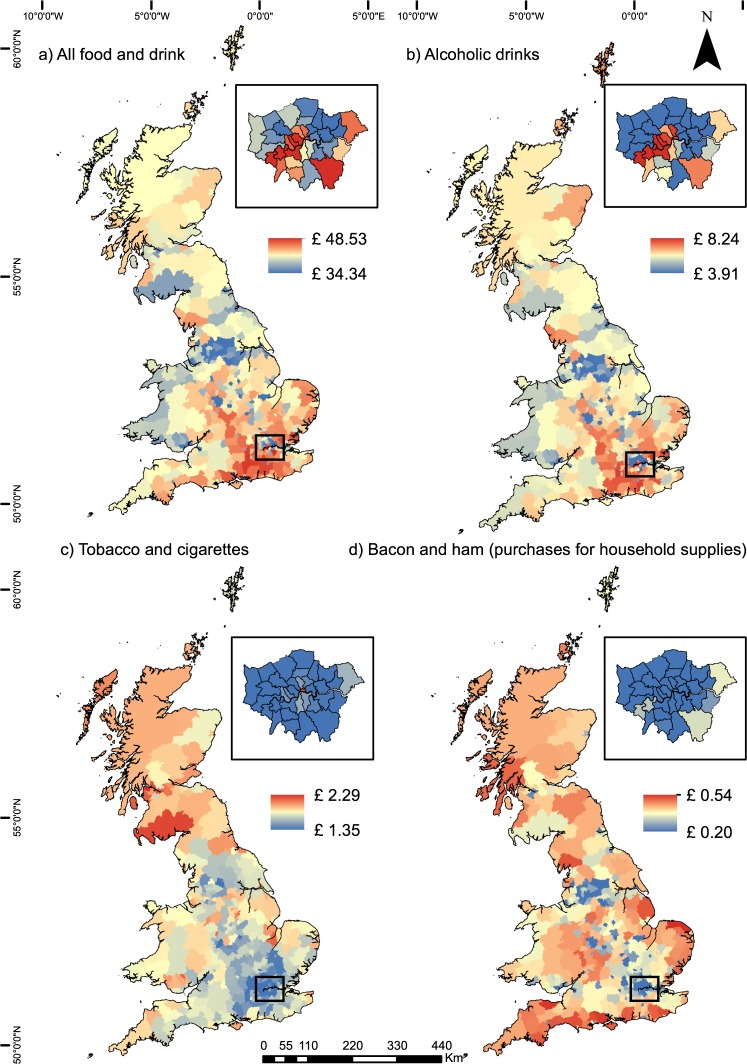
Estimated average weekly expenditure per person for 2012. Contains National Statistics data © Crown copyright and database right 2018. Contains OS data © Crown copyright and database right 2018.

Once the model is complete, GIS expenditure datasets may be created by joining the expenditure tables with spatial boundaries. Figure 2 shows examples of selected datasets for the year 2012. Cumulative categories are generated by summing the appropriate individual COICOP categories (e.g. all food and drink: Fig. 2a, alcoholic drinks: Fig. 2b, tobacco and cigarettes: Fig. 2c) whilst individual COICOP categories can also be mapped (e.g. bacon and ham purchased for household supplies: Fig. 2d). For visualisation and integration with other datasets, the GIS vector shapefiles may be converted to a spatial grid of data cells in a similar manner to other spatial datasets (e.g. James *et al*.^38^).

## Data Records

The local level expenditure datasets described in this article are publicly and freely available through Figshare^39^.

## Technical Validation

The validation of microsimulation models has received much attention in the literature due to the dangers of using incorrect model data to inform policy^40,41^. Validation of microsimulation models presents a substantial challenge since detailed spatial microdata are seldom available – in fact it can be argued that if such data were available the microsimulation process would be redundant. There are a variety of methods available for validation, broadly categorised as internal validation (ensuring the model makes sense in reality given the input data) and external validation (ensuring the model coincides with external reality).

### Internal validation

Internal validation is the most common form of microsimulation model evaluation and is the process of comparing the model’s output against data that are internal to the model itself^23^. We carried out internal validation in a similar manner to Lovelace, *et al*.^26^, by calculating for each LAD the correlation between the aggregate counts from the constraint variables and those generated in our spatial microsimulation. In our models, the results were affirmative; the lowest correlation for a single zone for all years was 0.9876 and in many cases was perfect (at least with an approximation to 4 decimals). The high correlation coefficients throughout give us confidence that the microsimulation process has worked correctly and common issues such as empty cells^42^ and incorrectly specified constraint variables are not present.

However, internal validation needs to be viewed in context as IPF microsimulation always converges towards the optimal solution for known constraint variables: it is the unknown cross-tabulations and target variables that we are trying to simulate with spatial microsimulation, so external validation should also be used^42^.

### External validation

In addition to internal validation, we use two methods for external model validation: a) by comparing the simulation results at the aggregate level with estimates from a dataset external to the model, and b) by aggregating-up the small area estimates provided by the simulation to compare the results with expenditure data that is provided at higher geographies.

Previous studies have shown that there are relationships between socio-economic status/deprivation and expenditure on certain commodities. Total food expenditure and consumption of fruit and vegetables has been shown to be greater in more affluent households^3,43^ whilst smoking prevalence is often greater in more deprived areas^44^ and among those of lower socio-economic status^45^. As such, we explored the relationship between our LAD level estimates of product expenditure and deprivation as measured by an external dataset. We used the Index of Multiple Deprivation (IMD) (https://www.gov.uk/government/statistics/english-indices-of-deprivation-2015), the official measure of relative deprivation for local authorities in England. IMD is based on seven different domains of deprivation: income deprivation; employment deprivation; education, skills and training deprivation; health deprivation and disability; crime; barriers to housing; and services and living environment deprivation. Whilst some of the domains are similar in nature to the constraints used in the microsimulation model (e.g. income deprivation), there are no common datasets between the IMD and the microsimulation, with metrics calculated in different ways. Furthermore, many of the IMD domains are completely absent from the microsimulation (e.g. crime, health deprivation and disability) and vice-versa, resulting in a minimal risk of circularity when exploring relationships. As the majority of datasets used in the IMD were collected in 2012 and 2013 we use the 2013 microsimulation results to test for a correlation against the IMD. As the IMD is a ranked dataset, we use the Spearman’s test of rank correlation, with the results shown in Table 12.

**Table 12:** Correlation between IMD and microsimulation estimate for categories previously identified as being correlated with deprivation.

	Spearman’s Rho	P-value
All food and drink	0.69	<0.01
Fruit and vegetables	0.69	<0.01
Tobacco and cigarettes	−0.33	<0.01

All correlations were significant with a strong positive correlation between IMD and estimates of ‘all food and drink’ and ‘fruit and vegetable’ expenditure, suggesting that expenditure on these categories is less in more deprived areas, as found by previous research^3,43^. Conversely, there is a negative correlation between tobacco and cigarette expenditure and IMD, suggesting expenditure is greater in more deprived areas, in agreement with previous studies^44^. These results are encouraging, suggesting the model is accurately capturing variation in expenditure for different product categories at small area level.

**Fig. 3:**
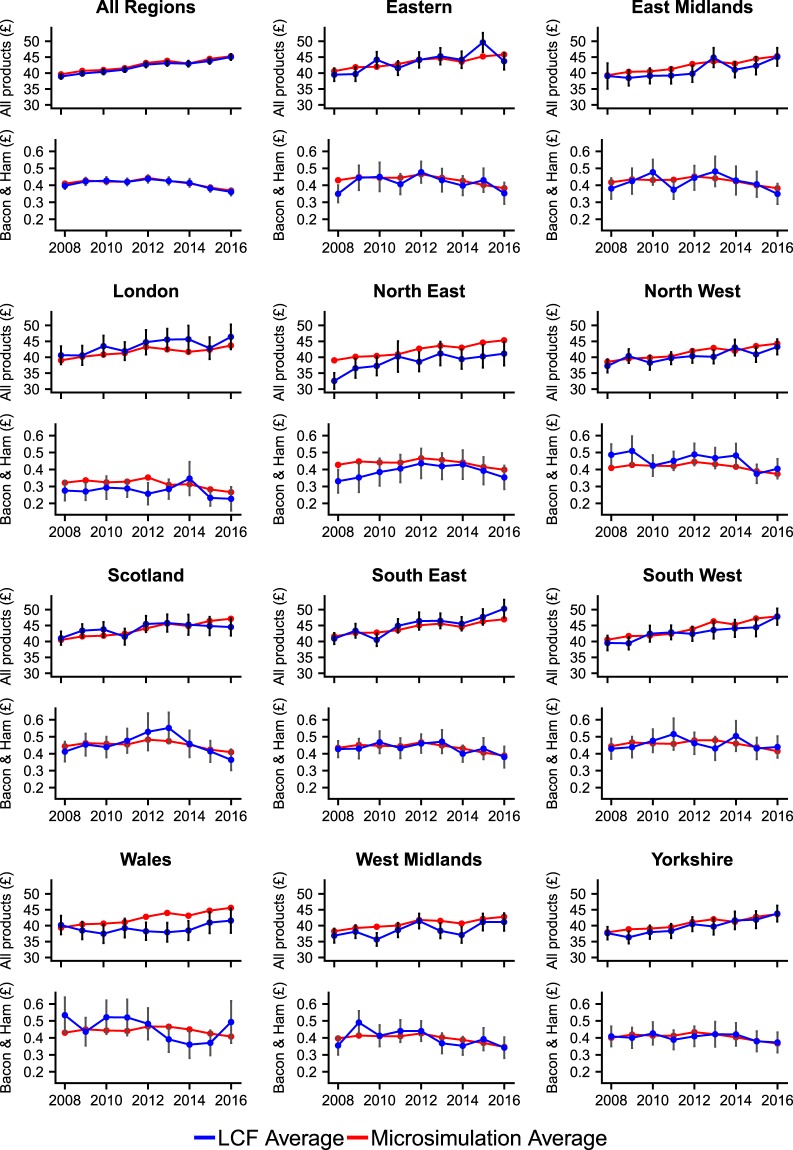
Estimates of average weekly expenditure per person using regionally averaged LCF survey values (blue line) and microsimulation aggregates (red line).

As noted previously, the LCF is geocoded to a limited extent, with information provided on which of the 12 regions each individual resides. This information is used by the Department for Environment, Food and Rural Affairs (DEFRA) to estimate the expenditure of products at the regional level, as published in the annual Family Food report series^16^. As this regional geographic information is not used in the microsimulation model other than for adjusting for relative regional price levels, this presents a useful form of validation by comparing the aggregated microsimulation results at the regional level to the corresponding values estimated directly from the LCF. Whilst both methods estimate the same parameter (expenditure by region), they are generated in completely different ways. The LCF averaging approach (as used by DEFRA^16^) takes the average weighted expenditure of surveyed individuals in each region, whilst the microsimulation approach generates a synthetic population with every individual assigned an expenditure profile which is then aggregated to the regional level. Figure 3 shows the results of the microsimulation model (aggregated to the regional level) alongside the corresponding values from the LCF, including error bars (±1.96 standard errors). Results are shown for a grouped category (all products) and an individual COICOP category (bacon and ham for household supplies). Corresponding 2012 maps for these categories are shown in Fig. 2.

Figure 3 demonstrates that the aggregated microsimulation results shows good correspondence with the LCF regional averages for both grouped categories (all products) and single COICOP categories (bacon and ham). Reassuringly, the majority of microsimulation estimates fall within the 95% confidence limit of the original LCF averages and the general trends in the LCF time series are reflected in the aggregated microsimulation results. This suggests that the simulated microdata correspond well to the ‘real world’ survey data. Whilst some regions such as the South East show a very close alignment between the aggregated microsimulation results and the LCF regional averages, others are not as closely aligned (e.g. North East). There are a number of possible reasons for these differences observed, including localised factors not accounted for by the microsimulation constraint variables or insufficient detail of input information. For example, the relative regional consumer price levels used to account for spatial differences in product pricing are only available for 2010^37^ and 2016^36^ and only at the aggregate level for regions within England (Table 11). It should also be noted that the confidence limits referred to here for the LCF only cover sampling errors and not non-sampling errors (systematic errors and random errors)^32^, and as such this uncertainty in the LCF should also be considered when comparing the estimates.

The internal and external validations presented here suggest that the microsimulation estimates of expenditure are capturing real differences. As such, we believe spatial microsimulation techniques of the type described in this paper hold great potential benefits for a range of disciplines including economics, retail geography and public health. Whilst this study focusses on Great Britain, the framework here could be applied to any location with the appropriate data sources.

## Usage Notes

The expenditure estimates produced in this study are based on data from the LCF, Census and other official sources. Therefore, the outputs provided are subject to any biases or errors contained in the source datasets. Household surveys such as the LCF have the potential to suffer from issues such as non-response from specific groups (e.g. low income households^46^) and measurement error^32^, especially in relation to products consumed away from home^47^ and alcohol^48^. Whilst the ONS has a robust sampling, weighting and quality control framework for the LCF^32^, the end user should be aware of the potential biases and errors, especially when considering specific commodities or socio-demographic categories.

## Supplementary Information

### ISA-Tab metadata file

Download metadata file

### Supplementary information

Supplementary File 1

## Data Availability

The R (version 3.3.1; https://www.r-project.org) code developed for production of the expenditure datasets are publicly and freely available through Figshare^39^. The script is internally documented to both explain its purpose and, when required, guiding the user through its customisation.

## References

[1] Kearney J (2010). Food consumption trends and drivers. Philos. Trans. R. Soc. London B Biol. Sci..

[2] Department for Environment Food and Rural Affairs (DEFRA). Health and Harmony: The Future for Food, Farming and the Environment in a Green Brexit. (Department for Environment Food and Rural Affairs (DEFRA), 2018).

[3] Venn D, Dixon J, Banwell C, Strazdins L (2018). Social determinants of household food expenditure in Australia: the role of education, income, geography and time. Public Health Nutr.

[4] Darmon N, Drewnowski A (2008). Does social class predict diet quality?. Am. J. Clin. Nutr.

[5] Pampel FC, Krueger PM, Denney JT (2010). Socioeconomic disparities in health behaviors. Annu. Rev. Sociol..

[6] Zhang H, Wang J, Martin W (2018). Factors affecting households’ meat purchase and future meat consumption changes in China: a demand system approach. J. Ethn. Foods.

[7] Department for Environment Food and Rural Affairs & Office for National Statistics. *Living Costs and Food Survey, 2008.* [*data collection*]. *3rd Edition*, 10.5255/UKDA-SN-6385-1 (2011).

[8] Department for Environment Food and Rural Affairs & Office for National Statistics. *Living Costs and Food Survey, 2009.* [*data collection*]. *4th Edition*, 10.5255/UKDA-SN-6655-1 (2011).

[9] Department for Environment Food and Rural Affairs & Office for National Statistics. *Living Costs and Food Survey, 2010.* [*data collection*]. *2nd Edition*, 10.5255/UKDA-SN-6945-2 (2012).

[10] Department for Environment Food and Rural Affairs & Office for National Statistics. *Living Costs and Food Survey, 2011.* [*data collection*]. *2nd Edition*, 10.5255/UKDA-SN-7272-2 (2013).

[11] Department for Environment Food and Rural Affairs & Office for National Statistics. *Living Costs and Food Survey, 2012.* [*data collection*]. *3rd Edition,*10.5255/UKDA-SN-7472-3 (2016).

[12] Department for Environment Food and Rural Affairs & Office for National Statistics. *Living Costs and Food Survey, 2013.* [*data collection*]. *2nd Edition*, 10.5255/UKDA-SN-7932-2 (2018).

[13] Department for Environment Food and Rural Affairs & Office for National Statistics. *Living Costs and Food Survey, 2014.* [*data collection*]. *3rd Edition*, 10.5255/UKDA-SN-7992-4 (2018).

[14] Department for Environment Food and Rural Affairs & Office for National Statistics. *Living Costs and Food Survey, 2015–2016.* [*data collection*]. *3rd Edition*, 10.5255/UKDA-SN-8210-5 (2018).

[15] Department for Environment Food and Rural Affairs & Office for National Statistics. *Living Costs and Food Survey, 2016–2017.* [*data collection*]. *3rd Edition*, 10.5255/UKDA-SN-8351-1 (2018).

[16] Department for Environment Food and Rural Affairs (DEFRA). *Family Food 2016/17*. (DEFRA, 2018).

[17] Orcutt GH (1957). A new type of socio-economic system. Rev. Econ. Stat..

[18] World Health Organization (WHO). *Noncommunicable diseases: progress monitor 2017*. (World Health Organization (WHO), 2017).

[19] Gerber, P. J. *et al*. *Tackling climate change through livestock: a global assessment of emissions and mitigation opportunities*. (Food and Agriculture Organization of the United Nations (FAO), 2013).

[20] De Sy V (2015). Land use patterns and related carbon losses following deforestation in South America. Environ. Res. Lett..

[21] Larson NI, Story MT, Nelson MC (2009). Neighborhood environments: disparities in access to healthy foods in the US. Am. J. Prev. Med..

[22] Bélanger, A. & Sabourin, P. *Microsimulation and Population Dynamics*. (Springer, 2017).

[23] Lovelace, R. & Dumont, M. *Spatial microsimulation with R*. (CRC Press, 2016).

[24] Clark, S., Birkin, M., Heppenstall, A. & Rees, P. Using 2011 Census data to estimate future elderly health care demand. In *The Routledge Handbook of Census Resources, Methods and Applications: Unlocking the UK 2011 Census*. (ed. Stillwell, J.) (Routledge, 2016).

[25] Nelissen JHM (1991). Household and education projections by means of a microsimulation model. Econ. Model..

[26] Lovelace R, Ballas D, Watson M (2014). A spatial microsimulation approach for the analysis of commuter patterns: from individual to regional levels. J. Transp. Geogr..

[27] Ballas D (2005). SimBritain: a spatial microsimulation approach to population dynamics. Popul. Space Place.

[28] Birkin, M. & Clarke, M. Spatial microsimulation models: a review and a glimpse into the future. In *Population dynamics and projection methods* (eds Stillwell, J. & Clarke, M.) (Springer, 2011).

[29] Lomax N, Smith A (2017). Microsimulation for demography. Aust. Popul. Stud..

[30] Fienberg SE (1970). An iterative procedure for estimation in contingency tables. Ann. Math. Stat..

[31] Lomax N, Norman P (2016). Estimating population attribute values in a table: “get me started in” iterative proportional fitting. Prof. Geogr..

[32] Office for National Statistics (ONS). *Living Costs and Food Survey Technical Report for survey year: April 2015 to March 2016*. (Office for National Statistics (ONS), 2017).

[33] Office for National Statistics (ONS). *Methodology used to produce household projections for England: 2016-based*. (Office for National Statistics (ONS), 2018).

[34] Office for National Statistics (ONS). *2011 Census analysis: What Does the 2011 Census Tell Us About People Living in Communal Establishments*. (Office for National Statistics (ONS), 2015).

[35] Office for National Statistics (ONS). *A guide to labour market statistics*. (Office for National Statistics (ONS), 2018).

[36] Office for National Statistics (ONS). *Relative regional consumer price levels of goods and services, UK: 2016*. (Office for National Statistics (ONS), 2018).

[37] Office for National Statistics (ONS). *UK Relative Regional Consumer Price levels for Goods and Services for 2010*. (Office for National Statistics (ONS), 2010).

[38] James WHM (2018). Gridded birth and pregnancy datasets for Africa, Latin America and the Caribbean. Sci. Data.

[39] James, W. H. M., Lomax, N. & Birkin, M. Local level estimates of food, drink and tobacco expenditure for Great Britain. *figshare*, 10.6084/m9.figshare.c.4300919 (2019). 10.1038/s41597-019-0064-z PMC6513822 31086192

[40] Edwards KL, Clarke GP (2009). The design and validation of a spatial microsimulation model of obesogenic environments for children in Leeds, UK: SimObesity. Soc. Sci. Med..

[41] Clarke M, Holm E (1987). Microsimulation methods in spatial analysis and planning. Geogr. Ann. Ser. B, Hum. Geogr.

[42] Edwards KL, Clarke GP, Thomas J, Forman D (2011). Internal and external validation of spatial microsimulation models: small area estimates of adult obesity. Appl. Spat. Anal. Policy.

[43] Giskes K, Turrell G, Patterson C, Newman B (2002). Socioeconomic differences among Australian adults in consumption of fruit and vegetables and intakes of vitamins A, C and folate. J. Hum. Nutr. Diet..

[44] Wise J (2014). UK survey confirms link between deprivation and smoking. BMJ Br. Med. J..

[45] De Vries H (1995). Socio-economic differences in smoking: Dutch adolescents’ beliefs and behaviour. Soc. Sci. Med..

[46] Office for National Statistics (ONS). *Limitations*. (Office for National Statistics (ONS), 2014).

[47] Fiedler JL, Yadav S (2017). How can we better capture food away from Home? Lessons from India’s linking person-level meal and household-level food data. Food Policy.

[48] Ramstedt M (2010). How much alcohol do you buy? A comparison of self‐reported alcohol purchases with actual sales. Addiction.

